# Comparison of different normalization strategies for the analysis of glomerular microRNAs in IgA nephropathy

**DOI:** 10.1038/srep31992

**Published:** 2016-08-24

**Authors:** Clemens L. Bockmeyer, Karen Säuberlich, Juliane Wittig, Marc Eßer, Sebastian S. Roeder, Udo Vester, Peter F. Hoyer, Putri A. Agustian, Philip Zeuschner, Kerstin Amann, Christoph Daniel, Jan U. Becker

**Affiliations:** 1 Department of Nephropathology, Friedrich Alexander University (FAU) Erlangen-Nuremberg, Erlangen, Germany; 2 Institute of Pathology, Hannover Medical School, Hannover, Germany; 3 Institute of Pathology, University Hospital of Cologne, Cologne, Germany; 4 Children’s Hospital, Pediatrics II, University of Duisburg-Essen, Essen, Germany; 5 Department of Nephrology, Hannover Medical School, Hannover, Germany

## Abstract

Small nucleolar RNAs (snoRNAs) have been used for normalization in glomerular microRNA (miRNA) quantification without confirmation of validity. Our aim was to identify glomerular reference miRNAs in IgA nephropathy. We compared miRNAs in human paraffin-embedded renal biopsies from patients with cellular-crescentic IgA-GN (n = 5; crescentic IgA-GN) and non-crescentic IgA-GN (n = 5; IgA-GN) to mild interstitial nephritis without glomerular abnormalities (controls, n = 5). Laser-microdissected glomeruli were used for expression profiling of 762 miRNAs by low-density TaqMan arrays (cards A and B). The comparison of different normalization methods (GeNormPlus, NormFinder, global mean and snoRNAs) in crescentic IgA-GN, IgA-GN and controls yielded similar results. However, levels of significance and the range of relative expression differed. In median, two normalization methods demonstrated similar results. GeNormPlus and NormFinder gave different top ranked reference miRNAs. Stability ranking for snoRNAs varied between cards A and B. In conclusion, we suggest the geometric mean of the most stable reference miRNAs found in GeNormPlus (miR-26b-5p), NormFinder (miR-28-5p) and snoRNAs (RNU44) as reference. It should be considered that significant differences could be missed using one particular normalization method. As a starting point for glomerular miRNA studies in IgA nephropathy we provide a library of miRNAs.

IgA nephropathy is the most common glomerulonephritis worldwide^1^. Currently histopathological analysis of renal biopsies is considered the gold standard for diagnosis and contributes to prognostication and therapeutic decision making^2^. miRNAs have been suggested as new biomarkers for renal disease activity to improve tissue diagnostics^3^. miRNAs act as post-transcriptional regulators of gene expression and recent studies have shown that it is feasible to measure gene expression of miRNAs in FFPE tissue^4^,^5^. They have been investigated extensively in renal diseases in general (reviewed in ref. 6) and in IgA nephropathy (reviewed in ref. 7) in particular. Whole tissue gene expression analysis demonstrated a higher intrarenal expression of miR-146a and miR-155-5p in patients with IgA nephropathy compared to healthy controls^8^,^9^. miR-21-5p was found to be up-regulated in laser-microdissected glomeruli of patients with IgA nephropathy and its inhibition prevented fibrogenic activation in podocytes^10^. The same group demonstrated a down-regulation of miR-223 in glomeruli with endocapillary proliferation^11^, however endocapillary proliferation was not clearly defined in this study.

Despite the recent surge of studies on miRNAs in kidney diseases, a profound assessment of reference miRNAs for proper quantification of miRNA expression by RT-qPCR has not been provided yet. To the best of our knowledge, no comprehensive study has identified suitable miRNA reference genes in whole tissue or in laser-microdissected glomeruli from paraffin tissue. It has even been shown that expression levels of small-nucleolar RNAs (snoRNAs) such as RNU48 and RNU44, which are suggested as references by manufacturers, are regulated in neoplastic diseases like breast cancer and head and neck squamous cell carcinoma^12^ making their use as reference miRNAs highly questionable. We are not aware of any studies about the stability of snoRNAs in non-neoplastic renal diseases.

The aim of the study was (i) to provide a comprehensive data set of glomerular miRNAs expression in native renal biopsies of IgA-GN on a cohort reflecting the full spectrum of glomerular tuft pathology (ii) to identify the most stably expressed miRNAs as a reference by proper normalization strategies (iii) to compare different normalization strategies for the identification of differentially expressed miRNAs in “active, cellular” crescentic IgA-GN vs. IgA-GN vs. controls. This provides basic information for further studies about the pathogenesis of IgA nephropathy especially to identify novel molecular markers for disease activity in urinary, serum and tissue diagnostics.

## Materials and Methods

### Patients and biopsies

Biopsies from 15 different patients were selected from the archive of the Institute of Pathology, Hannover Medical School: Five with IgA-GN without crescents, five with crescentic IgA-GN (only with active, cellular crescents, >20% of glomeruli involved) and five mild intersititial nephritis without glomerular abnormalities as controls. The former two cohorts reflected the full spectrum of glomerular tuft findings in IgA-GN not only according to the Oxford classification but also including crescentic forms and regarding IgG and IgM deposits (see Table 1). Importantly, none of the patients received any medication at the time of biopsy. All biopsies were examined with our standard diagnostic protocol and scored according to the MEST criteria of the Oxford classification^2^. Detailed information for every biopsy is given in Table 1. Other forms of renal and systemic diseases were excluded.

### Clinical data and serology

Clinical data included blood pressure, proteinuria (none, non-nephrotic, nephrotic), hematuria, hemoglobin, leukocyte count, thrombocyte count, serum-creatinine and eGFR (MDRD formula; *Schwartz* formula, if age <18 years; see Table 2). We could not find any significant differences in serum creatinine and eGFR between the cohorts in pairwise comparisons using Steel-Dwass tests. Thus, we should have reasonably controlled any secondary effects of uremia on glomerular tuft miRNA expression.

### Glomerular microdissection and miRNA high throughput analysis

Between 150 to 500 glomerular cross sections from paraffin-embedded biopsies (cut at a thickness of 3 μm) were deparaffinized for 30 sec. in xylole, stained for 2 sec. in haemalaun (Mayers Haemalaun, Merck, Germany) and washed in sterile aqua ad injectabilia (Ampuwa, Plastipur, Fresenius, Germany). From dried sections only open capillary loops were microdissected, carefully excluding the crescents, global and segmental sclerosed glomeruli (mmi^®^ CellCut Plus^®^ Laser Microdissection System, Eching, Germany).

RNA was quantified as described previously^13^,^14^. Briefly, isolated RNA was dissolved in 10 μl DEPC water. Thereof 3.0 μl were reverse transcribed using the Multi-Scribe-based High Capacity Kit and Megaplex RT stem-loop primer pool A and B, Version 2.0 (Applied Biosystems, Foster City, CA, USA), enabling RNA-specific cDNA synthesis for 762 different small RNA species including snoRNAs (snRNU6 (U6, RNU6A), RNU48 and RNU44) and several tRNA derived fragments^15^. For the sake of convenience all measured tRNA species are termed as miRNAs according to the old nomenclature. Preamplification and quantification by TaqMan low density arrays (TLDA) were performed as described^16^. Sequences and accession numbers of defined endogenous reference genes as well as miRNAs with significantly different expression are given in Supplementary Table 1.

### Evaluation of miRNA expression data

Amplification curves and C_q_ values (quantitation cycle) were generated with the software RQ manager 1.2 (Applied Biosystems). Threshold values for all TLDA cards were set at 0.25. Amplification curves for every reaction were inspected visually and underwent a stringent quality control. C_q_ values of amplification curves that could not be trusted were set as C_q_ = 45. Baseline values were adjusted if necessary^17^. Based on a preamplification procedure following megaplex reverse transcription and TLDA we defined C_q_ values of >32.0 as off-scale C_q_ values (reactions that are too high to be trusted)^18^. Out of 381 we excluded 201/321 miRNAs in Pool A/B for further analysis, when a C_q_ value of >32.0 in more than 5 out of 15 examined samples was obtained. For the remaining 240 miRNAs we used the recommended pragmatic approach to replace all missing C_q_ values (reactions that did not yield any C_q_-value) and off-scale C_q_ values by C_q_ = 33^17^.

### Defining candidate reference genes

A typical normalization study using geNormPlus or NormFinder uses only data for highly expressed candidate reference genes. Therefore we only used those miRNAs with not more than one off-scale Cq-value or missing Cq-value. Furthermore, following the algorithm of Wotschofsky *et al.*^19^ we included only miRNAs that met the criteria of non-regulated miRNAs. First all C_q_ values had to be converted into relative quantities (RQ). We used the highest C_q_ value as calibrator (highest C_q_ value in one patient sample – each C_q_ value of a specific miRNA in this patient sample). Then all values had to be standardised by calculating the ratio of RQ divided by the arithmetic mean of RQ from all miRNAs in the same patient sample. In a second step these transformed RQ values were used for another global normalization by calculating the ratio of transformed RQ divided by the arithmetic mean of RQ from all patient samples of one miRNA. Next, we analyzed the standard deviation of RQ for each miRNA in all patient samples, the expression differences as well as the mean fold change differences between subgroups. Finally, those nonregulated miRNAs were selected as candidate reference genes for geNormPlus/NormFinder, which passed 2 out of the following 3 criteria: (i) standard deviation <0.0003 (Pool A) as well as <0.00003 (Pool B), (ii) p-value for expression differences >0,7 in non-parametric Steel-Dwass tests and (iii) mean fold change between −0.00003 and 0.00003 (Pool A) as well as between −0.000015 and 0.000015 (Pool B). Standard deviation and mean fold changes were calculated with Microsoft Excel (Version 12.2.8).

### Defining normalization factors

Four different normalization factors were defined by calculating the geometric mean of C_q_ values^20^ from each of the recommended reference miRNAs: Recommendation of (i) geNormPlus, (ii) NormFinder, (iii) global mean normalization method, (iv) two most stable snoRNAs or (v) all three snoRNAs provided by the manufacturer.

The commercially available geNormPlus algorithm in qBase^plus^, version 3.0 (http://www.biogazelle.com/qbaseplus Biogazelle, Zwijnaarde, Belgium) is based on calculating the average pairwise variations of each candidate reference genes compared to all candidate reference genes^21^. Step by step the gene with the highest variation (highest M-Value) was excluded from the list of candidate genes and the variation analysis is repeated with the remaining candidates. In the end, the remaining candidate gene with the lowest M-value is identified as the best reference gene^20^. For the identification of the optimal number of reference genes geNormPlus provides the following algorithm: Differences in the ratio of the average pairwise variation of normalization factors (geometric mean C_q_ value)^20^ of consecutive candidate reference genes starting with the two most stable genes (n) and the addition of the next most stable gene (n + 1) until all genes have been added suggest the number of optimal reference genes. As a general guideline, the benefit of using an extra reference gene is limited as soon as the difference in the ratio (Vn/n + 1) drops below the 0.15 threshold^22^.

The second normalization method we used was the NormFinder algorithm, which identifies reference genes via a simple interface (an Excel add-in available as a free download under http://moma.dk/normfinder-software)^23^,^24^, which analyses different subgroups (IgA-GN, crescentic IgA-GN and controls) independently. Hereby intragroup and intergroup variations are considered independently. The stability value for each gene is a measurement of the estimated systematic error when using this gene for normalization. The best 3 reference genes were chosen for calculating the normalization factor. For both algorithms the amplification efficiency was assumed as 100%. Imported C_q_ values were automatically transformed into relative expression data by the software^25^. Furthermore, we skipped those miRNAs belonging to the same miRNA-family (see miRNAVISA web interface^26^) and used only the best miRNA per family.

We also used the global mean normalization method. It calculates normalization factors based on the geometric mean of C_q_ values of all analyzed genes of one patient sample^18^,^27^. This method is recommended for experiments with more than 50 randomly distributed genes over all biological pathways.

Moreover, snoRNAs (RNU44, RNU48 and U6 snRNA) were used as a reference as recommended by the manufacturer. Relative expression was determined using the 2^−ΔCq^ method. ΔC_q_ was calculated as C_q_(target) – normalization factor.

### Statistical Analysis

For pairwise comparisons of continuous parameters between the cohorts we used the Steel-Dwass on JMP 9.0.2 (SAS Institute, Cary, NC, USA). Nonparametric tests are recommended in case of replaced missing or off-scale C_q_ values. P-values <0.05 were considered statistically significant in two-tailed tests. Median values, 25/75 percentiles (boxes) as well as 10/90 percentiles (whiskers) are shown in Figures with box plots. Median values as well as the quartiles are shown in Table 2. GraphPad Prism Version 5.0 (GraphPad Software Inc., La Jolla, CA, USA) was used for plotting of values.

The Spearman’s rank correlation coefficients were applied to calculate the association of values yielded from geNormPlus and NormFinder.

### Ethical Approval

All studies were carried out according to the latest revision of the Declaration of Helsinki (http://www.wma.net/en/30publications/10policies/b3/index.html) and were approved by the Ethics Committee of Hannover Medical School (number 2241-2014), hereby it is documented that informed consent was obtained prospectively from all subjects.

## Results

### Reference miRNAs in studies related to non-neoplastic kidney diseases

First we performed a literature search in PubMed for articles published between January 2008 and December 2014, with the search terms “microRNA(s), micro-RNA(s) or miRNA(s)” and “kidney, renal, (IgA-) glomerulonephritis or (IgA-)nephropathy”. We focused on manuscripts about human, rat or mouse non-neoplastic renal tissues. Papers about urinary and serum analysis using spiked references as well as *in vitro* studies were excluded.

70 publications reported miRNA expression by RT-qPCR in renal tissue (18 times human, 39 times mouse, 13 times rat) and used small nuclear, nucleolar or ribosomal RNAs as well as mRNAs for normalization, namely snRNU6 (32 times), RNU48 (6 times), RNU87 (5 times), snoRNA202 (5 times), 5srRNA (5 times), 18srRNA (4 time), RNU6B (3 times) as well as others (GAPDH, RNU19, RNU44, RNU19, snoRNA 135, snoRNA 234) without confirming their validity for normalization (see Supplementary Table 2, including literature). miR-193a and miR-16 were the only two reference miRNAs, which were properly identified as stably expressed in polycystic kidney disease and diabetic nephropathy, respectively^28^,^29^. Five publications used laser-microdissection for glomerulus-specific analysis^10^,^11^,^30^,^31^,^32^.

### Inclusion of 240 microRNAs for further analysis

**Figure 1:**
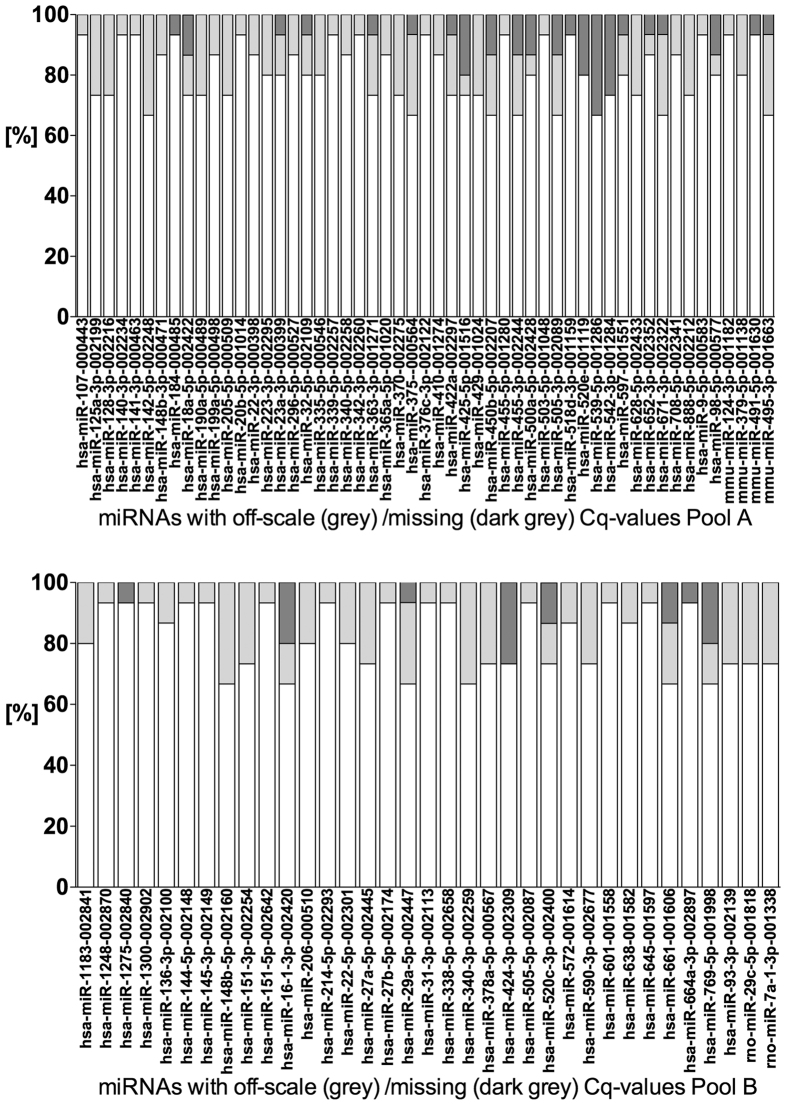
miRNAs with off-scale (grey) and missing (dark grey) C_q_ values are shown for quality control. Percentages for 15 patient samples are shown for each miRNA. Grey boxes are for off-scale and dark grey boxes for missing C_q_ values. Out of these miRNAs seven were also defined as candidate reference miRNAs (miR-20b, miR-342-3p, miR-1275, miR-145-3p, miR-151-5P, miR-27b-5p, miR-601). However, none of these were ranked within the top 15 reference genes by geNormPlus or NormFinder.

Two-hundred and fourty (180 from Pool A and 60 from Pool B) out of 762 measured miRNAs (32%) were included for further analysis according to our algorithm described in the methods section. C_q_ values of these 240 miRNAs are provided in a supplementary excel file (Supplementary Table 3). According to Stahlberg *et al.* a defined amount of off-scale C_q_ or missing C_q_ values within these 240 miRNAs was accepted^17^. We decided to use this threshold for exclusion of miRNAs in order not to miss any relevant data. As shown in Fig. 1 we found 53 (Pool A)/35 (Pool B) miRNAs with off-scale C_q_ or missing C_q_ values in our final analysis. Among the 2685/900 further analyzed C_q_ values there were 39 (1.5%)/17 (1.9%) missing C_q_ values and 112 (4.2%)/78 (8.7%) off-scale C_q_ values in Pool A/B. miR-708-5p demonstrated two off-scale C_q_ values in controls, consistent with a significantly lower glomerular expression of this miRNA in controls. Beside this miRNA neither other significantly expressed target genes nor snoRNAs had any off-scale or missing C_q_ value.

### Stability analysis of candidate reference genes

**Figure 2:**
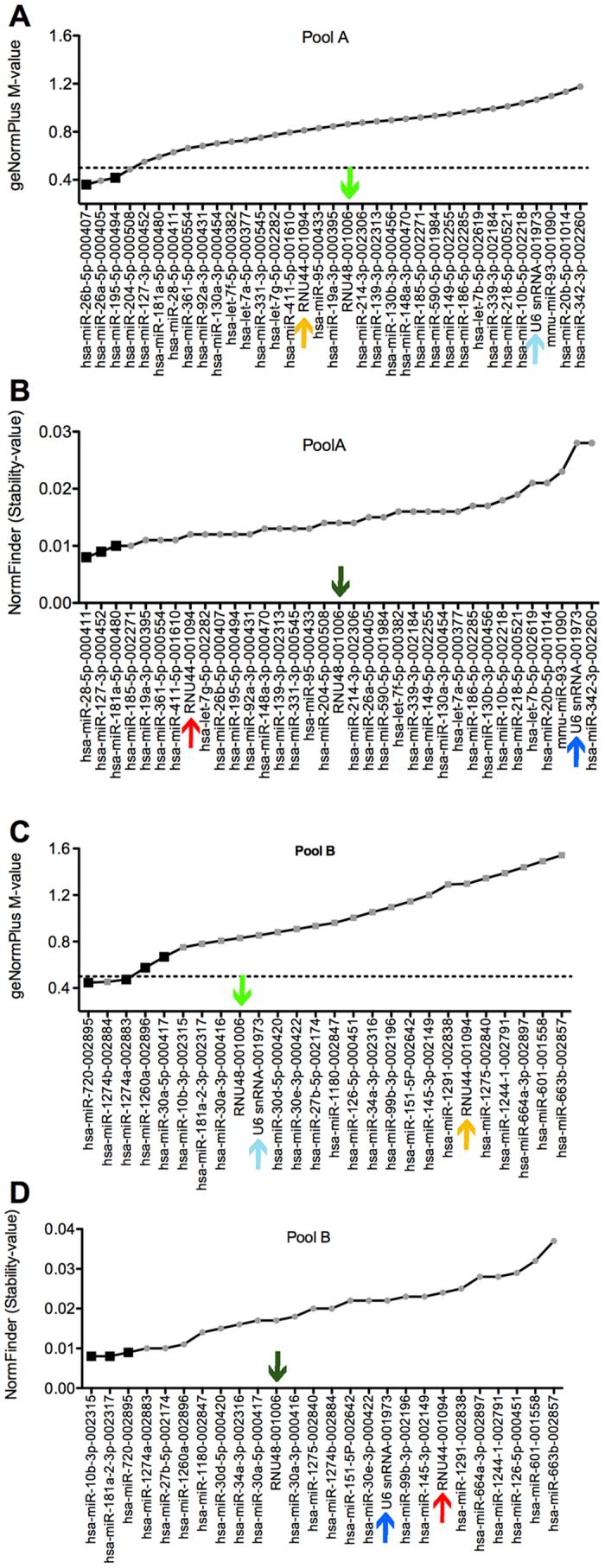
Results of geNormPlus and NormFinder. geNormPlus analysis shows the calculation of the average expression stability M-value of all candidate reference genes determined by RT-qPCR (**A,C**). Genes with the highest M-value have the least stable expression, while the genes with the lowest M-value have the most stable expression. The x-axis presents the ranking of reference genes in order of decreasing stability from left to the right. High stability is defined by an M-value of <0.5 as indicated by the dotted line. Chosen reference miRNAs are highlighted by black squares. By analogy NormFinder Stability-values are listed in an ascending order (**B,D**). Small-nucleolar RNAs (snoRNAs such as RNU48, RNU44 and U6snRNA) are indicated by coloured arrows. snoRNAs are indicated by coloured arrows.

**Figure 3:**
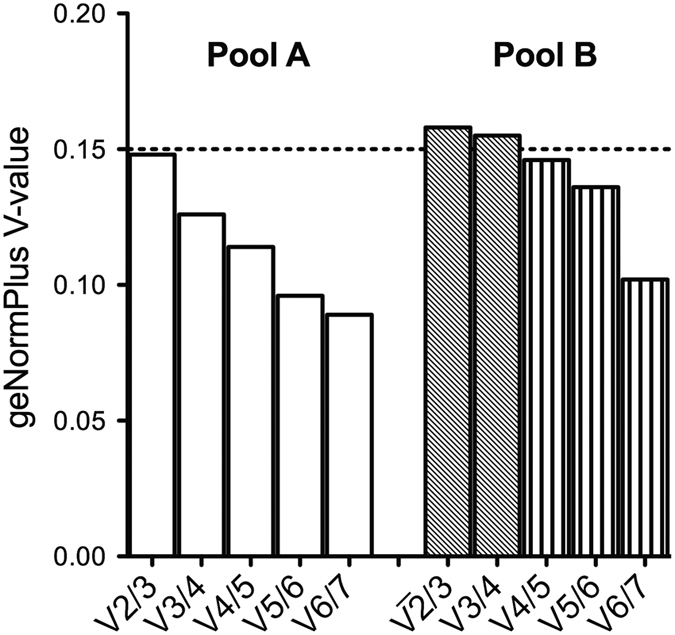
Calculation of the optimal number of reference genes for normalization by geNormPlus. geNormPlus calculates the optimal number of reference genes taking into account the variable V as the average pairwise variation between two sequential candidate reference miRNAs. The dotted line illustrates the cut-off value 0.15. In our cohort the optimal number of reference genes was two (V2/3) for Pool A (white boxes) and four (V4/5) for Pool B (grey boxes indicate a V-value above the cut-off).

For the computational approach of geNormPlus and NormFinder we used 35 miRNAs (Pool A) and 26 miRNAs (Pool B) as candidate reference genes (Fig. 2) defined by the algorithm described in the method section. Out of these, miR-20b, miR-342-3p (Pool A) and miR-1275, miR-145-3p, miR-151-5p, miR-27b-5p, miR-601 (Pool B) had a maximum of one off-scale/missing C_q_ value (Fig. 1). GeNormPlus and NormFinder analysis recommended different reference genes. GeNormPlus recommended miR-26b-5p and miR-195-5p (Pool A) as well as miR-720, miR-1274a, miR-1260a and miR-30a-5p (Pool B) as the best combinations (Fig. 2). miR-26a and miR-1274b were excluded, because they belong to the same miRNA-family/tRNA derived fragment and had a higher stability-value in the NormFinder analysis. The pairwise variation V2/3 calculated to indicate the optimal number of reference genes required for normalization yielded a value of 0.148 in Pool A, which was already below 0.15 (Fig. 3). Thus, based on geNormPlus, a combination of the best two reference genes is stable enough for Pool A and the addition of another miRNA would not improve normalization accuracy. In Pool B V4/5 yielded a value of 0.146 (Fig. 3). Therefore the best four reference genes are suggested as normalization factor.

**Figure 4:**
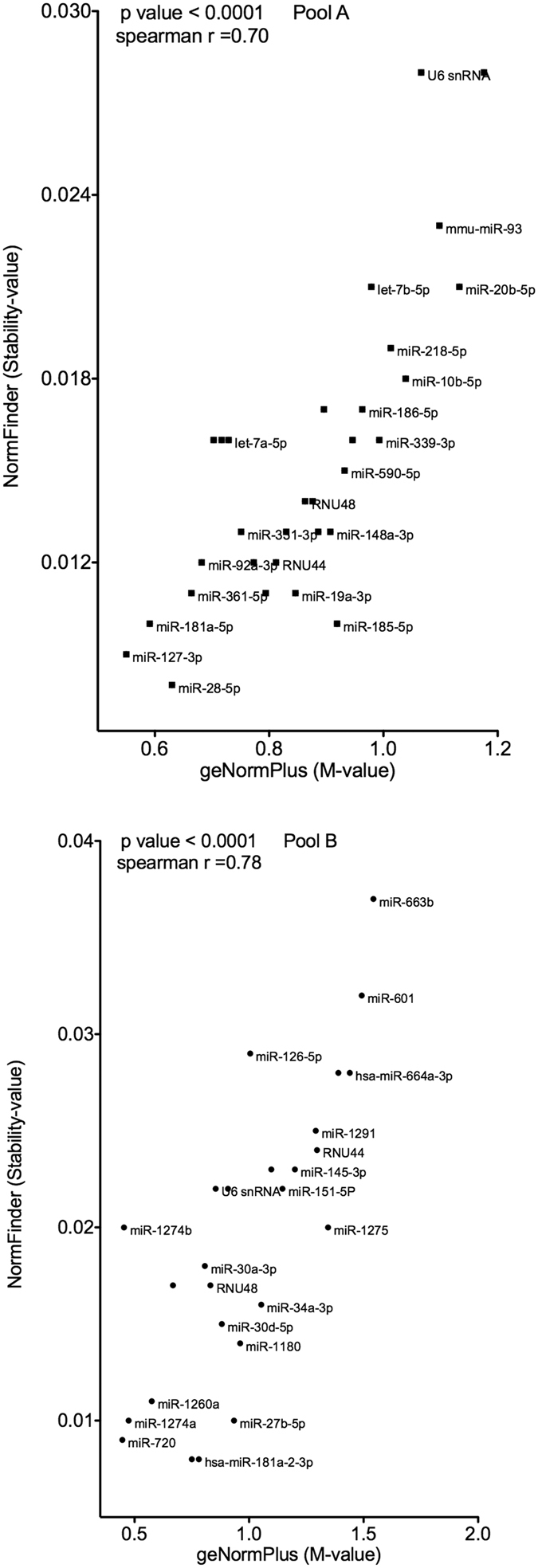
Direct correlation of geNormPlus and NormFinder. Spearman’s rank correlation coefficients indicate a high consistency between those two algorithms. For the sake of legibilty not all data points are labeled.

In contrast, NormFinder identified miR-28-5p, miR-127-3p and miR-181a-5p for Pool A as well as miR-10b-3p, miR-181a-2-3p and miR-720 for Pool B as the most stable miRNAs (Fig. 2). There was a significant correlation of stability-values comparing geNormPlus and NormFinder (Fig. 4).

### snoRNAs as endogenous reference transcripts

**Figure 5:**
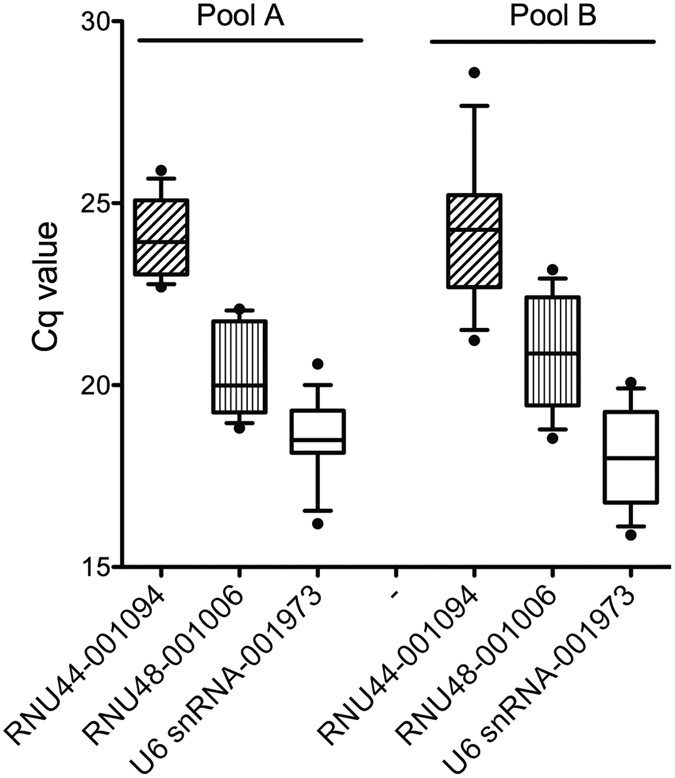
Variation in expression of snoRNAs on card A and B. Quantification cycle (Cq) values were plotted for each sample. There was a higher variation for all snoRNAs on card B.

Interestingly the order of stability-values for snoRNAs differed between TLDA cards A and B (indicated by coloured arrows in Fig. 2). RNU44 was ranked 16^th^ (Pool A)/21^st^ (Pool B) by geNormPlus, 8^th^ (Pool A)/20^th^ (Pool B) by NormFinder. RNU48 was ranked 19^th^ (Pool A)/9^th^ (Pool B) by geNormPlus, 18^th^ (Pool A)/11^th^ (Pool B) by NormFinder. snRNU6 was ranked 32^nd^ (Pool A)/10^th^ (Pool B) by geNormPlus, 34^th^ (Pool A)/17^th^ (Pool B) by NormFinder. The comparison of C_q_ values for snoRNAs revealed a higher interquartile range of all analyzed snoRNAs on card B (Fig. 5). Due to this discrepancy we decided to analyze our data with the best two snoRNAs as well as by all three snoRNAs as references.

### miRNAs with significantly different expression

**Figure 6:**
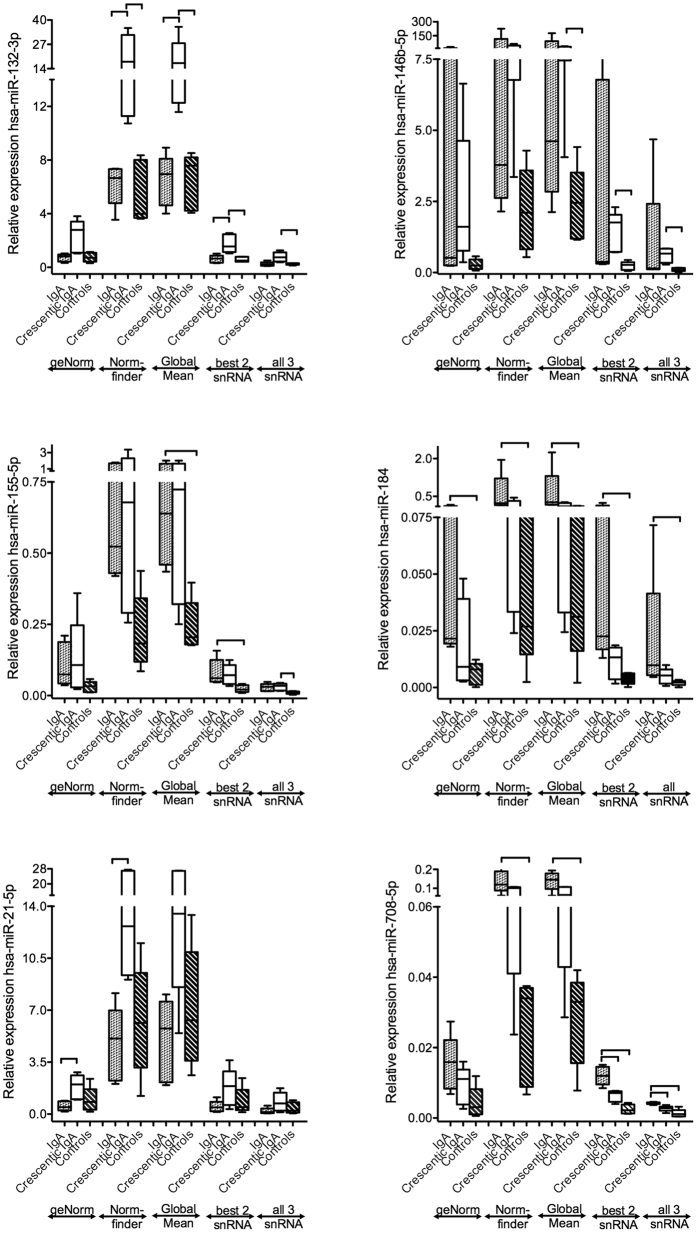
Effects of different normalization approaches on the expression of miRNAs in IgA-GN. Quantitative differences of six significantly different expressed miRNAs are shown in dependence from different normalization factors. Relative expression levels were calculated using the following normalization methods: geNormPlus (miR-26b, miR-195-5p) NormFinder (miR-28-5p, miR-127-3p, miR-181a-5p), global Mean, best two snRNAs (RNU44, RNU48) and all 3 snRNAs (RNU44, RNU48, snRNU6). Four miRNAs (miR-132-3p, miR-146-5p, miR-184, miR-708-5p) demonstrated high consistency throughout different normalization methods. Significant differences are illustrated by horizontal lines as P values < 0.05 of the nonparametric pairwise ranking test (Steel-Dwass test for multiple comparison analysis). Median values, 25/75 percentiles (grey dotted boxes, IgA-GN; white, crescentic IgA-GN; striped, controls) as well as 10/90 percentiles (whiskers) are shown.

In order to demonstrate the value of our reference miRNAs, we examined our small cohort for differences between tufts from crescentic and non-crescentic IgA-GN. Active crescents have long been considered an important indicator of inflammatory activity and have been suggested for inclusion in the Oxford classification^33^. For the detection of differentially expressed microRNAs we analyzed 240 target miRNAs with four different normalization methods: Recommendation of (i) geNormPlus (geometric mean of miR-26b-5p and miR-195-5p (Pool A) as well as of miR-720, miR-1274a, miR-1260a and miR-30a-5p (Pool B)), (ii) NormFinder (geometric mean of miR-28-5p, miR-127-3p and miR-181a-5p (Pool A) as well as miR-10b-3p, miR-181a-2-3p and miR-720 (Pool B)), (iii) global mean normalization method, (iv) two most stable snoRNAs (geometric mean of RNU44 and RNU48 (Pool A) as well as RNU48 and snRNU6 (Pool B)) or (v) geometric mean of all three snoRNAs provided by the manufacturer. We compared three subgroups: IgA-GN, crescentic IgA-GN and controls. A total of 12 miRNAs (Pool A 10 miRNAs, Pool B 2 miRNAs) showed differential expression. Of these miRNAs six are shown in Fig. 6 and another six in the Supplementary Fig. All normalization methods yielded similar results for these 12 miRNAs, however with different levels of significance. In particular miR-132-3p, miR-30b-5p and miR-30c-5p were strongly expressed compared to reference miRNAs. The global mean method and the NormFinder method demonstrated the highest rates of concordance. Four miRNAs (miR-132-3p, miR-146-5p, miR-184, miR-708-5p) demonstrated high persistence throughout different normalization methods (at least 3 out of 5 normalization methods with similar significant results). Comparing all 12 significantly expressed miRNAs in median two normalization methods demonstrated similar significant results. The global mean method demonstrated the highest number of significant results when comparing crescentic IgA-GN vs. IgA-GN. Specifically, miR-132-3p, miR-146-5p and miR-27a-5p were elevated in crescentic IgA-GN vs. controls as well as miR-155-5p, miR-184 and miR-708-5p in IgA-GN vs. controls. In addition, miR-132-3p, miR-125b-5p and miR-21-5p were significantly elevated in crescentic IgA-GN vs. IgA-GN, whereas miR-132-3p and miR-184 demonstrated highest persistency throughout different normalization methods. On the other hand, miR-708-5p and let7c were significantly elevated in IgA-GN vs. crescentic IgA-GN as well as miR-30c-5p, miR-30b-5p, hsa-miR-505-5p in controls vs. crescentic IgA-GN.

## Discussion

In this study we provide the first RT-qPCR-array-based identification of appropriate reference genes for miRNA expression studies in laser-microdissected glomeruli of patients from a wide spectrum of IgA-GN. It is widely accepted that there is no “one fits all” reference gene. Instead one has to determine which genes exhibit the greatest stability for the experimental conditions under examination. Results from mRNA expression studies based on non-validated reference genes can be misleading^34^,^35^. As an overview on reference miRNAs used in non-neoplastic renal tissue expression studies we compiled data from different studies. This Pubmed search showed that snoRNAs such as snRNU6, RNU48, RNU87, snoRNA202 and ribosomal RNA such as 5srRNA and 18srRNA are most frequently used as reference transcripts, however without any validation. A similar overview for reference miRNAs in serum, plasma, blood monocytes and blood of cardiovascular, non-neoplastic hepatic or pulmonary diseases failed to show consensus reference snoRNAs^36^. Except for snRNU6 and RNU48, none of these snoRNAs are provided on the TLDA card we used. Most importantly in our study snRNU6 and RNU48 were not among the most stably expressed by geNormPlus and NormFinder algorithms. *M*-values were higher than expected (0.8–1.2) and they were even ranked differently on card A and card B. This might be due to intercard variability of snoRNAs as described by others^37^. In mRNA studies *M*-values should be less than 0.5^20^. Thus, other transcripts than these snoRNAs seem to be better suited as references for glomerular miRNA studies in IgA nephropathy.

For identification of reference miRNAs in laser-microdissected glomeruli of patients with IgA nephropathy we measured 762 miRNAs, analyzed 240 miRNAs and included 61 miRNAs as candidate reference genes. Compared to all 2,500 (including all −5p and −3p sequences) human “high confidence” miRNAs reported in the miRBase release from June 2014 (release 21)^38^ these were 30%, 9.6% and 2.4%, respectively. We compared the stability of miRNAs between crescentic IgA-GN, IgA-GN in order to identify the best references for broad spectrum of IgA nephropathy.

In order to provide a set of reference miRNAs one could suggest the intersection of the best 15 reference genes from both algorithms geNormPlus and NormFinder (miR-10b-3p, miR-1260a, miR-127-3p, miR-1274a, miR-181a-5p, miR-181a-2-3p, miR-195-5p, miR-26b-5p, miR-28-5p, miR-30a-3p, miR-30a-5p, miR-30d-5p, miR-361-5p, miR-720, miR-92a-3p). The ranking of the M-value (geNormPlus) compared to the stability-value (NormFinder) of those 15 miRNAs was not identical. However, considering (i) the high correlation between both algorithms, (ii) that each algorithm employs a different mathematical approach and (iii) that the confidence of the respective results will vary depending on the innate variability of the dataset, we suggest a set of strong reference miRNAs. Furthermore, these 15 reference miRNAs are associated with independent cellular processes, increasing the confidence that any observed differences are accurate. Lastly, they were highly expressed and did not have any off-scale or missing C_q_ values.

Several miRNAs like miR-181a-5p, miRNA-195-5p, miR-720, and miR-26b-5p have been described in other studies. *In situ* hybridisation stained miR-181a-5p in tubular epithelial cells, especially close to the juxtaglomerular apparatus. Whole tissue analysis of human nephrectomy specimens has shown that the tubular expression of miR-181a-5p is associated with renin mRNA expression^39^,^40^. This is in accordance with the finding of an aberrant renal miR-181a-5p expression in mouse^41^ and human^40^ hypertensive kidneys. In our small cohort without any differences in arterial blood pressure miR-181a-5p was stably expressed in lasermicrodissected glomeruli with IgA nephropathy, indicating that miR-181a-5p is not primarily affected by IgA-GN or neighboring crescents. Due to its stable glomerular expression, differences in whole tissue analysis of miR-181a-5p seem to be due to an altererd tubulointerstitial or vascular expression.

miRNA-195-5p and miR-720 seem to be relevant in diabetic glomerulopathy. Under *in vitro* high-glucose conditions inhibition of miRNA-195-5p protected mesangial cells from apoptosis and induces mesangial cellular proliferation^42^, in contrast, miR-195-5p mediated podocyte apoptosis^43^. Furthermore, miR-195-5p was identified as an inhibitor of sirtuin 1 (Sirt1) in diabetic nephropathy^44^. Histone deacetylase Sirt1 ameliorated diabetic nephropathy via multiple mechanisms^45^,^46^,^47^,^48^. miR-720 was associated with microalbuminuria in human diabetic nephropathy^49^. Moreover, miR-720 was up-regulated and miR-26b-5p down-regulated in isolated glomeruli of murine diabetic nephropathy^42^. Therefore miRNA-195-5p, miR-26b-5p and miR-720 should be used with caution or completely avoided as reference transcripts in diabetic subjects. In our cohorts, diabetes was excluded on clinical and histological grounds.

Another two miRNAs were analyzed in whole tissue analysis. miR-30a-3p was decreased in acute cellular rejection^50^. miR-30d-5p was down-regulated in progressive kidney disease from diabetic and/or hypertensive nephropathy^51^. TGF-ß treatment of podocytes *in vitro* resulted in a diminished expression of all miR-30 family members^52^. Therefore one could speculate that miR-30 family members play a role in the complex network of podocytopathia-associated up-regulation of TGF-ß^53^. For the other stably expressed miRNAs no studies in non-neoplastic kidney tissue are available. In summary, we are not aware of any study arguing against the use of those 15 glomerular reference miRNAs in IgA nephropathy.

Using several different normalization strategies, we aimed to minimize the limitations of any specific method while capitalising on the unique strengths of each approach. As a key result from our study all methods provided similar results. On average about 2 out of 5 different normalization methods provided even similar levels of significance, despite the small cohort sizes. Compared to the global mean method, which is often referred to as the gold standard, the NormFinder method as well as the snoRNA method (two most stable snoRNAs) provided very similar results. However, there are still two questions remaining: How many differences between cohorts would remain undetected with an inappropriate normalization strategy? And how should we identify the most appropriate normalization strategy? In order to answer these questions, we compared our favorite normalization approaches: (A) best **two** of geNormPlus (miR-195-5p, miR-26b-5p) and NormFinder (miR-127-3p, miR-28-5p), (B) best **one** of geNormPlus (miR-26b-5p), NormFinder (miR-28-5p) and snoRNA (RNU44) and (C) best **one** of geNormPlus (miR-26b-5p), best **two** of NormFinder (miR-127-3p, miR-28-5p) and best **one** of snoRNA (RNU44). As shown in the Table 3 (A, B, C) there were minor differences between those three normalization approaches, however we suggest the normalization approach (B) due to persistent detection of significance levels for miRNAs, when compared to different normalization methods.

Some differentially expressed miRNAs in histological variants of IgA nephropathy have been shown to regulate distinct pathways in renal diseases. miR-21-5p was up-regulated in crescentic IgA-GN using the normalization method of geNormPlus and NormFinder, however, in contrast to the study of Bao *et al.*^10^ we did not detect any difference in glomeruli of IgA-GN vs. controls. Unfortunately Bao *et al.* did not comment on the presence of crescents. They identified up-regulated glomerular miR-21-5p in 20 patients with IgA nephropathy using snRNU6^10^. Based on our findings, it appears questionable to use only the non-validated snRNU6 as a reference. The differences between the study of Bao *et al.* and this study could be due to the different normalization strategies used, missing information about active crescents in their cohort and due to the small size of our cohort. Moreover, Bao *et al.* did not comment on segmental or global glomerulosclerosis in their study cohort. This would have been important, because in mice a loss of miR-21-5p is associated with accelerated glomerulosclerosis^54^. In our cohort of crescentic IgA-GN all cases demonstrated segmental glomerulosclerosis.

miR-132-3p was up-regulated 3-fold in crescentic IgA-GN compared to both controls and IgA-GN. This is in line with miR-132-3p upregulation during transition between acute and fibrotic injury in a mouse model of folic acid-induced kidney injury and fibrosis^55^. Furthermore, miR-132-3p was highly increased in rat kidneys with hypertension and cardiac hypertrophy and seems to play a role in the Renin-Angiotensin-II-system^56^. In our study cohort we excluded patients on any medication including angiotensin-receptor blockers. One interesting target of miR-132-3p is Sirt1, which protected endothelial cells and enhances mesangial cell survival^57^. Sirt1 also regulated PGC-1a activity, which plays an essential role for maintenance of podocyte mitochondrial function^58^. Counterregulatory efforts upon injury in IgA nephropathy of one or even all-glomerular cell types (endothelium, mesangial cells and podocytes) could explain the dramatic up-regulation of miR-132-3p.

Two miRNAs, miR-148b and let-7b, which seem to mediate the aberrant O-glycosylation process of IgA and that can differentiate IgA nephropathy patients in serum tests^59^,^60^, were not significantly expressed in our cohort. In other diseases like in acute leukemia or gastric cancer it has been shown that serum levels of miRNA expression were not the same as in tissue samples^61^,^62^. This underscores the need to show the presence of a miRNA in the affected tissue in order to establish this miRNA as a biomarker. To this end, our study provides broad spectrum of miRNAs expressed in glomeruli of IgA nephropathy and controls. For future cost- and time-efficient analyses, we suggest to focus on those 240 miRNAs with high glomerular expression given in Supplementary Table 3. They could facilitate future large-scale studies looking into ancillary miRNA biomarkers in IgA nephropathy. We are not aware of any biomarker studies combining glomerular, serum and urine miRNA quantification in correlation with clinical and histological parameters. With the small number of patients in our study, which was aimed at providing an indispensable methodological base for future studies, we cannot provide such details yet. Moreover, due to the exclusion of miRNAs with Cq values >32 in more than 5 out of 15 samples we might not have been able to detect significant neo-expression or loss of miRNA expression in individual cohorts. It was necessary to follow our stringent algorithm for excluding miRNAs with off-scale/missing C_q_ values in order to focus on highly expressed miRNAs. For further analysis of quite small, but perhaps biologically relevant differences of less expressed miRNAs and for the quality control of biomarker studies, we provide all C_q_ values from glomerular miRNAs in the Supplementary Table 3.

In summary we provide an in-depth ranking of reference miRNAs indispensable for accurate normalization and quantification of glomerular miRNA expression studies in IgA-GN. Based on our analysis we suggest miR-26b-5p (best according to geNormPlus) and miR-28-5p (best according to NormFinder) and one snoRNA (RNU44) as suitable reference genes choice for human glomerular miRNA quantification in IgA nephropathy. Nevertheless, individual experimental conditions, especially different species (mouse, rat etc.) have to be considered and similar studies to ours might have to be conducted to identify better-suited references.

## Additional Information

**How to cite this article**: Bockmeyer, C. L. *et al.* Comparison of different normalization strategies for the analysis of glomerular microRNAs in IgA nephropathy. *Sci. Rep.* **6**, 31992; doi: 10.1038/srep31992 (2016).

## Supplementary Material

Supplementary Information

Supplementary Dataset 1

## Figures and Tables

**Table 1:** All biopsies were examined with our standard diagnostic protocol for Immunohistological features and scored according to the MEST criteria of the Oxford classification.

Group	IgA-staining	IgG-staining	IgM-staining	M	E	S	T	C
IgA	+++	negative	+	1	0	0	0	0
IgA	++	negative	++	1	0	0	0	0
IgA	+++	+	negative	1	0	0	0	0
IgA	++	++	+	1	1	0	0	0
IgA	+++	+	trace	1	0	0	0	0
Crescentic IgA	++	+	+	1	1	1	0	1
Crescentic IgA	+++	trace	trace	1	1	1	0	1
Crescentic IgA	+++	trace	trace	1	1	1	0	1
Crescentic IgA	++	trace	trace	1	1	1	0	1
Crescentic IgA	++	++	++	1	1	1	0	1
Control	negative	negative	trace	0	0	0	0	0
Control	negative	negative	trace	0	0	0	0	0
Control	negative	negative	trace	1	0	0	0	0
Control	negative	negative	negative	0	0	0	0	0
Control	negative	negative	negative	0	0	0	0	0

The entire spectrum of IgA-GN regarding concurrent IgM and IgG deposits with the IgA codominance defining IgA-GN was covered. Furthermore, the full spectrums of glomerular tuft findings according to the criteria of the Oxford classification are presented in our cohort. M mesangial hypercellularity, E endocapillary hypercellularity, S segmental glomerulosclerosis, T tubular atrophy/interstitial fibrosis, C only active, cellular crescent.

**Table 2:** Clinical data of patients with IgA nephropathy (non-crescentic IgA-GN (IgA-GN) and cellular-crescentic IgA-GN (crescentic IgA-GN)) and controls. If no unit is given data are presented as total count, otherwise as median (25^th^/75^th^ percentile).

	IgA-GN; n = 5	Crescentic-IgA-GN; n = 5	Controls; n = 5
Female	2	3	4
Age at biopsy	47.0 (37.0/51.0)	46.0 (17.0/65.0)	37.0 (24.0/55.0)
Crescents per total glomeruli count (%)	0 (0/0)	44.4 (36.6/46.2)	0 (0/0)
Global glomerulosclerosis per total glomeruli count (%)	5.9 (5.9/6.3)	9.8 (0/10)	0 (0/0)
Arterial hypertension WHO-Grade			
0	3	2	3
1	1	2	1
2	1	0	0
3	0	1	0
n.d.	0	0	1
Proteinuria			
normal	0	0	2
<3.5 g/day	5	3	3
>3.5 g/day	0	2	0
n.d.	0	0	0
Microhematuria	5	4	2
Macrohematuria	1	1	0
Haemoglobin (g/dl)	13.1 (11.1/15.4)	10.9 (10.8/12.7)	12.5 (9.1/14.1)
Leukocytes (10^3^/μl)	6.8 (5.6/7.4)	8.1 (7.7/8.8)	8.8 (5.7/11.3)
Thrombocytes (10^3^/μl)	237 (192/257)	360 (292/401)	245 (166/484)
Serum-Creatinine (μmol/l)	84 (68/232)	107 (79/450)	273 (129/465)
eGFR MDRD (ml/min) (*Schwartz if age* <*18 years*)	88.8 (42.2/96.0)	54.7 (22.1/95.5)	18.0 (15.8/40.8)

Although serum creatinine of controls was higher than those in patients with IgA nephropathy, there were no significant differences. Furthermore, there were no significant differences for eGFR (Steel-Dwass test).

**Table 3:** Summary of different normalization methods (i–v) compared to three different suggested normalization approaches: (A) best **two** of geNormPlus (miR-195-5p, miR-26b-5p) and NormFinder (miR-127-3p, miR-28-5p), (B) best **one** of geNormPlus (miR-26b-5p), NormFinder (miR-28-5p) and snoRNA (RNU44) and (C) best **one** of geNormPlus (miR-26b-5p), best **two** of NormFinder (miR-127-3p, miR-28-5p) and best **one** of snoRNA (RNU44).

miRNA	Suggested normalization approaches			Different normalization methods				
	(A) Best two of geNormPlus (miR-195-5p, miR-26b-5p) and NormFinder (miR-127-3p, miR-28-5p)	(*B*) *Best one of geNormPlus* (*miR-26b-5p*)*, NormFinder* (*miR-28-5p*) *and snoRNA* (*RNU44*)	(C) Best one of geNormPlus (miR-26b-5p), best two of NormFinder (miR-127-3p, miR-28-5p) and best one of snoRNA (RNU44)	(i) geNorm Plus	(ii) Norm Finder	(iii) global mean	(iv) best 2 snoRNAs	(v) best 3 snoRNAs
**miR-132-3p**	**p < 0.05**	***p* < *0.05***	**p < 0.05**	**n.s.**	**p < 0.05**	**p < 0.05**	**p < 0.05**	**n.s.**
**miR-146b-5p**	**n.s.**	***p* < *0.05***	**p < 0.05**	**n.s.**	**p < 0.05**	**p < 0.05**	**p < 0.05**	**p < 0.05**
miR-155-5p	n.s.	*p* < *0.05*	p < 0.05	n.s.	n.s.	p < 0.05	p < 0.05	n.s.
**miR-184**	**p < 0.05**	***p* < *0.05***	**p < 0.05**	**p < 0.05**	**p < 0.05**	**p < 0.05**	**p < 0.05**	**p < 0.05**
miR-21	p < 0.05	*n.s.*	n.s.	p < 0.05	p < 0.05	n.s.	n.s.	n.s.
**miR-708-5p**	**n.s.**	***p* < *0.05***	**p < 0.05**	**n.s.**	**p < 0.05**	**p < 0.05**	**p < 0.05**	**p < 0.05**
Let-7c	n.s.	*n.s.*	p < 0.05	n.s.	p < 0.05	p < 0.05	n.s.	n.s.

p values are given for each normalization strategy. miRNAs with persistent detection of significance levels throughout different normalization methods (recommendation of (i) geNormPlus, (ii) NormFinder, (iii) global mean normalization method, (iv) two most stable snoRNAs or (v) all three snoRNAs) are bolt printed. The suggested approach B (italic letters) provides the best compromise for detection and non detection of significant differences compared to the results of five different normalization methods. n.s. non significant.
