# Supplementary material for: Comparison of different normalization strategies for the analysis of glomerular microRNAs in IgA nephropathy
Source: Sci Rep. 2016 Aug 24;6:31992. doi: 10.1038/srep31992 (PMC4995590; doi:10.1038/srep31992)
Supplement: Supplementary Information [file srep31992-s1.pdf]

Supplementary information

Comparison of different normalization strategies for the analysis of glomerular microRNAs in IgA nephropathy

Clemens L. Bockmeyer[1,2], Karen Säuberlich[3], Juliane Witting[3], Marc Eßer[3], Sebastian S. Roeder[1], Udo Vester[4], Peter F. Hoyer[4], Putri A. Agustian[5], Philip Zeuschner[3], Kerstin Amann[1], Christoph Daniel[1], Jan U. Becker[3]

[1] Department of Nephropathology, Friedrich Alexander University (FAU) Erlangen-Nuremberg, Germany.
[2] Institute of Pathology, Hannover Medical School, Hannover, Germany.
[3] Institute of Pathology, University Hospital of Cologne, Cologne, Germany.
[4] Children's Hospital, Pediatrics II, University of Duisburg-Essen, Essen, Germany.
[5] Department of Nephrology, Hannover Medical School, Hannover, Germany.

Corresponding author:

Clemens L. Bockmeyer, MD

Department of Nephropathology

Institute of Pathology

FAU Erlangen-Nuremberg

Krankenhausstraße 8-10

91054 Erlangen

Germany

clemens.bockmeyer@uk-erlangen.de or

bockmeyer@gmail.com

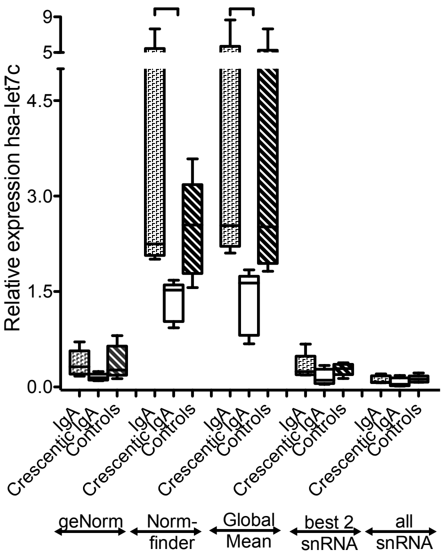
Relative expression hsa-let7c
9
5
4.5
3.0
1.5
0.0
IgA
Crescentic IgA
Controls
geNorm
Norm-finder
Global Mean
best 2 snRNA
all snRNA

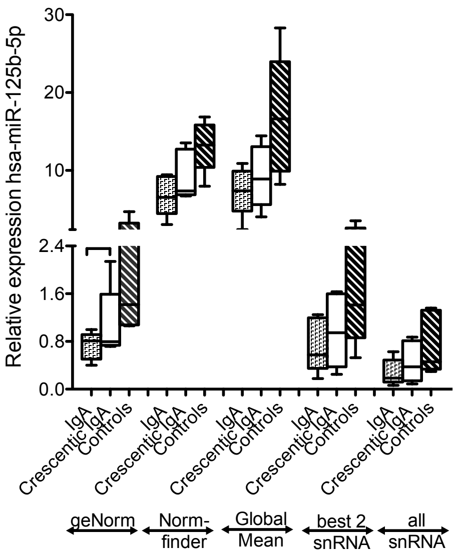
Relative expression hsa-miR-125b-5p
30
20
10
2.4
1.6
0.8
0.0
IgA
Crescentic IgA
Controls
geNorm
Norm-finder
Global Mean
best 2 snRNA
all snRNA

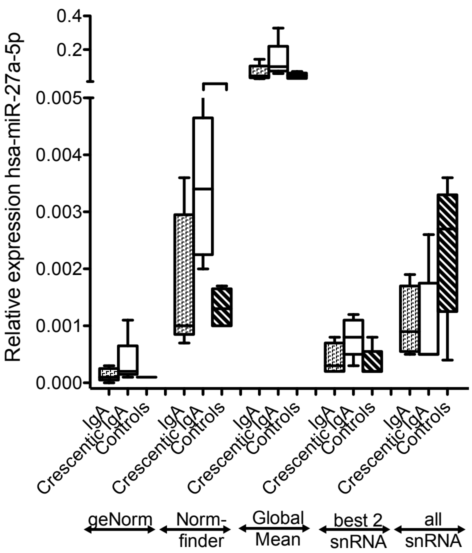
Relative expression hsa-miR-27a-5p
0.4
0.2
0.005
0.004
0.003
0.002
0.001
0.000
IgA
Crescentic IgA
Controls
geNorm
Norm-finder
Global Mean
best 2 snRNA
all snRNA

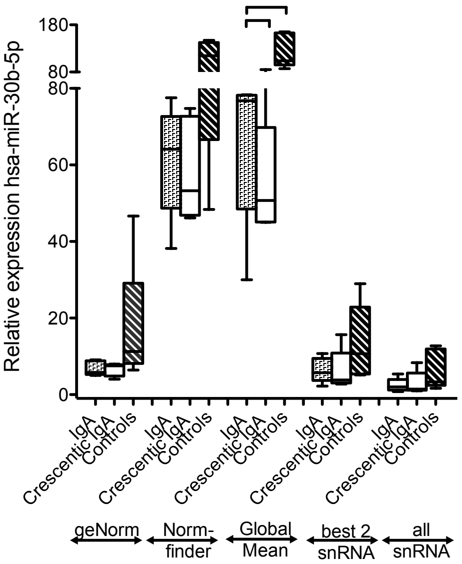
Relative expression hsa-miR-30b-5p
180
80
80
60
40
20
0
IgA
Crescentic IgA
Controls
geNorm
Norm-finder
Global Mean
best 2 snRNA
all snRNA

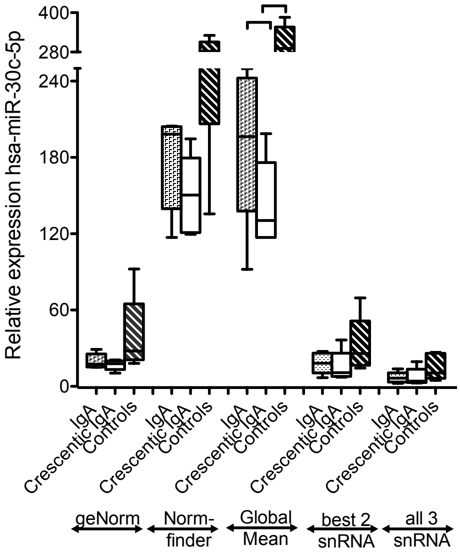
Relative expression hsa-miR-30c-5p
400
280
240
180
120
60
0
IgA
Crescentic IgA
Controls
geNorm
Norm-finder
Global Mean
best 2 snRNA
all 3 snRNA

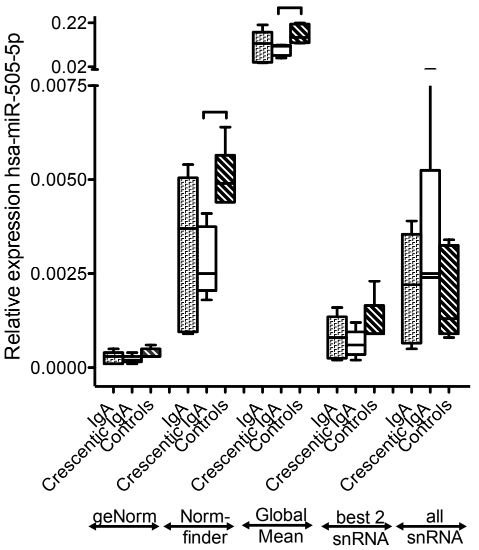
Relative expression hsa-miR-505-5p
0.22
0.02
0.0075
0.0050
0.0025
0.0000
IgA
Crescentic IgA
Controls
geNorm
Norm-finder
Global Mean
best 2 snRNA
all snRNA

Supplementary Figure

Another six miRNAs are shown in dependence of different normalization approaches in analogy to Fig. 6.. Most miRNAs demonstrated less consistency throughout different normalization methods. The best two snRNAs for normalization of miRNAs from Card A (let-7c, miR-125b-5p, miR-30b-5p, miR-30c-5p) were RNU44 and RNU48 in to contrast to Card B (miR-27a-5p, miR-505-5p) when RNU48 and snRNU6 was used.

Supplementary Table 1

Sequences and accession numbers of endogenous reference miRNAs suggested by geNormPlus and NormFinder. Furthermore, miRNAs with significantly different expression in IgA nephropathy are shown according to consistency throughout different normalization methods. All described miRNAs are human species.

Gene symbol	Previous gene symbol	Target sequence	Assay ID
**Intersection of the best 15 reference genes from geNormPlus and NormFinder**			
miR-10b-3p	miR-10b#	ACAGAUUCGAUUCUAGGGGAAU	002315
miR-1260a	miR-1260	AUCCCACCUCUGCCACCA	002896
miR-127-3p	miR-127	UCGGAUCCGUCUGAGCUUGGCU	000452
tRNA(thr) derived fragment	miR-1274A	GUCCCUGUUCAGGCGCCA	002883
miR-181a-5p	miR-181a	AACAUUCAACGCUGUCGGUGAGU	000480
miR-181a-2-3p	miR-181a-2#	ACCACUGACCGUUGACUGUACC	002317
miR-195-5p	miR-195	UAGCAGCACAGAAAUAUUGGC	000494
miR-26b-5p	miR-26b	UUCAAGUAAUUCAGGAUAGGU	000407
miR-28-5p	miR-28	AAGGAGCUCACAGUCUAUUGAG	000411
miR-30a-3p	miR-30a-3p	CUUUCAGUCGGAUGUUUGCAGC	000416
miR-30a-5p	miR-30a-5p	UGUAAACAUCCUCGACUGGAAG	000417
miR-30d-5p	miR-30d	UGUAAACAUCCCCGACUGGAAG	000420
miR-361-5p	miR-361	UUAUCAGAAUCUCCAGGGGUAC	000554
tRNA(thr) derived fragment	miR-720	UCUCGCUGGGGCCUCCA	002895
miR-92a-3p	miR-92a	UAUUGCACUUGUCCCGGCCUGU	000431
**Significantly different with high** consistency throughout different normalization methods			
miR-132-3p	miR-132	UAACAGUCUACAGCCAUGGUCG	000457
miR-146b-5p	miR-146b	UGAGAACUGAAUUCCAUAGGCU	001097
miR-184	miR-184	UGGACGGAGAACUGAUAAGGGU	000485
miR-708-5p	miR-708	AAGGAGCUUACAAUCUAGCUGGG	002341
**Significantly different miRNAs with medium** consistency throughout different normalization methods			
miR-155-5p	miR-155	UUAAUGCUAAUCGUGAUAGGGGU	002623
miR-21-5p	miR-21	UAGCUUAUCAGACUGAUGUUGA	000397
let7c	let7c	UGAGGUAGUAGGUUGUAUGGUU	000379
**Significantly different miRNAs with less** consistency throughout different normalization methods			
miR-125b-5p	miR-125b	UCCCUGAGACCCUAACUUGUGA	000449
miR-27a-5p	miR-27a#	AGGGCUUAGCUGCUUGUGAGCA	002445
miR-30b-5p	miR-30b	UGUAAACAUCCUACACUCAGCU	000602
miR-30c-5p	miR-30c	UGUAAACAUCCUACACUCUCAGC	000419
miR-505-5p	miR-505#	GGGAGCCAGGAAGUAUUGAUGU	002087

Supplementary Table 2

Overview of applied reference genes in publications between January 2008 and December 2014 about miRNAs related to non-neoplastic renal diseases. Studies on IgA-GN are highlighted bold printed.

Autor / Seniorautor	Topic	Titel	Journal	Year	Reference microRNA	Renal Tissue (species)
Kato/ Natarajan	Diabetic Nephropathy	Post-transcriptional Up-regulation of Tsc-22 by Ybx1, a Target of miR-216a, Mediates TGF-β-induced Collagen Expression in Kidney Cells	J. Biol. Chem	2010	18s rRNA	mouse
Putta/ Natarajan	Renal Fibrosis	Inhibiting microRNA-192 ameliorates renal fibrosis in diabetic nephropathy	J. Am. Soc. Nephrol	2012	18s rRNA	mouse
Patel/ Igarashi	Polycystic Kidney Disease	miR-17~92 miRNA cluster promotes kidney cyst growth in polycystic kidney disease.	PNAS	2013	18s rRNA and 5s rRNA	mouse
Kato/ Natarajan	Diabetic Nephropathy	TGF-beta activates Akt kinase through a microRNA-dependent amplifying circuit targeting PTEN	Nat Cell Biol.	2009	18s rRNA or 5s rRNA	mouse
Kato/ Natarajan	Diabetic Nephropathy	MicroRNA-192 in diabetic kidney glomeruli and its function in TGF-beta-induced collagen expression via inhibition of E-box repressors	PNAS USA	2007	5s rRNA	mouse
Kato/ Natarajan	Glomerular Fibrosis	A microRNA circuit mediates transforming growth factor-β1 autoregulation in renal glomerular mesangial cells	Kidney Int	2011	5s rRNA	mouse
Fu/Yi	Diabetic Nephropathy	Regulation of NADPH oxidase activity is associated with miRNA-25-mediated NOX4 expression in experimental diabetic nephropathy.	Am. J. Nephrol	2010	5s rRNA	rat
Liu/Liang	Hypertension	Renal medullary microRNAs in Dahl salt-sensitive rats: miR-29b regulates several collagens and related genes	Hypertension	2010	5s rRNA	rat
Bai/Chen	Aging /Development	miR-335 and miR-34a promote renal senescence by suppressing mitochondrial antioxidative enzymes.	J Am Soc Nephrol	2011	5s rRNA	rat
Lin/Wang	Physiology/ Anatomy	MicroRNA 802 stimulates ROMK channels by suppressing caveolin-1	J. Am. Soc. Nephrol	2011	GAPDH	mouse
Krupa/Fraser	Diabetic Nephropathy	Loss of MicroRNA-192 promotes fibrogenesis in diabetic nephropathy	J. Am. Soc. Nephrol	2010	miR-16 (TLDA)	human
Pandey/Gretz	Polycystic Kidney Disease	Microarray-based approach identifies microRNAs and their target functional patterns in polycystic kidney disease	BMC Genomics	2008	miR-193a	rat
Dweep/ Gretz	Polycystic Kidney Disease	Parallel analysis of mRNA and microRNA microarray profiles to explore functional regulatory patterns in polycystic kidney disease: using PKD/Mhm rat model	PLoS One	2013	miR-193a	rat
Mladinov/ Laing	Physiology/ Anatomy	MicroRNAs contribute to the maintenance of cell-type-specific physiological characteristics: miR-192 targets Na+/K+-ATPase β1	Nucleic Acids Res	2013	nicht angegeben	rat
Chau/Duffield	Renal Fibrosis	MicroRNA-21 promotes fibrosis of the kidney by silencing metabolic pathways.	Sci. Transl. Med.	2012	RNU19	human
Anglicheau/ Suthanthiran	Renal Allograft Rejection	MicroRNA expression profiles predictive of human renal allograft status	PNAS USA	2009	RNU44	human
Wang/Szeto	Hypertension	Intrarenal expression of miRNAs in patients with hypertensive nephrosclerosis.	Am J Hypertens	2010	RNU48	human
**Wang/Szeto**	**IgA nephropathy**	**Intrarenal expression of microRNAs in patients with IgA nephropathy.**	**Lab. Invest**	**2010**	**RNU48**	**human**
**Wang/Szeto**	**IgA nephropathy**	**Elevated levels of miR-146a and miR-155 in kidney biopsy and urine from patients with IgA nephropathy**	**Dis. Markers**	**2011**	**RNU48**	**human**
Zhou/Illei	Lupus nephritis	miR-150 promotes renal fibrosis in lupus nephritis by downregulating SOCS1	J. Am. Soc. Nephrol	2013	RNU48	human
Brennan/ Godson	Renal Fibrosis	Lipoxins attenuate renal fibrosis by inducing let-7c and suppressing TGFβR1	JASN	2013	RNU48	human
Lu/Szeto	Lupus nephritis	Glomerular and tubulointerstitial miR-638, miR-198 and miR-146a expression in lupus nephritis	Nephrology (Carlton)	2012	RNU48	human (frozen, laser-microdissection)
Denby/ Baker	Renal Fibrosis	miR-21 and miR-214 are consistently modulated during renal injury in rodent models.	Am. J. Pathol	2011	RNU87	rat
Li/ Lin	Diabetic Nephropathy	MiR-124 is related to podocytic adhesive capacity damage in STZ-induced uninephrectomized diabetic rats	Kidney Blood Press Res	2013	RNU87	rat
Ramdas/ Baker	Renal Fibrosis	Canonical transforming growth factor-β signaling regulates disintegrin metalloprotease expression in experimental renal fibrosis via miR-29.	Am. J. Pathol.	2013	RNU87 / snRNU6	mouse/rat

Autor / Seniorautor	Topic	Titel	Journal	Year	Reference microRNA	Renal Tissue (species)
Wang/ Kantharidis	Diabetic Nephropathy	miR-200a Prevents renal fibrogenesis through repression of TGF-beta2 expression.	Diabetes	2011	RNU87 or snoRNA135	mouse/rat
Wang/ Kantharidis	Renal Fibrosis	Transforming growth factor-β1-mediated renal fibrosis is dependent on the regulation of transforming growth factor receptor 1 expression by let-7b.	Kidney Int	2014	RNU87 or snoRNA135	mouse/rat
Wang/Quigg	Diabetic Nephropathy	MicroRNA-377 is up-regulated and can lead to increased fibronectin production in diabetic nephropathy	FASEB J	2008	snoRNA 234/ U18	human/mouse
Ichii/Kon	Chronic inflammation	Altered expression of microRNA miR-146a correlates with the development of chronic renal inflammation.	Kidney Int	2012	snoRNA202	mouse
Bhatt/Dong	Acute Kidney Injury	MicroRNA-34a is induced via p53 during cisplatin nephrotoxicity and contributes to cell survival	Mol Med	2010	snoRNA202	mouse
Godwin/ Iacomini	Acute Kidney Injury	Identification of a microRNA signature of renal ischemia reperfusion injury	PNAS USA	2010	snoRNA202	mouse
Shapiro/ Iacomini	Acute Kidney Injury	MicroRNA Expression Data Reveals a Signature of Kidney Damage following Ischemia Reperfusion Injury	PLoS one	2011	snoRNA202	mouse
Wie/Dong	Acute Kidney Injury	Targeted Deletion of Dicer from Proximal Tubules Protects against Renal Ischemia-Reperfusion Injury	J. Am. Soc. Nephrol	2010	snoRNA202	mouse
Marrone/Ho	Genetic renal disease	MicroRNA‑17~92 is required for nephrogenesis and renal function	J. Am. Soc. Nephrol	2014	snoRNA234/ Rps17	mouse
Dai/Huang	Lupus nephritis	Comprehensive analysis of microRNA expression patterns in renal biopsies of lupus nephritis patients	Rheumatol. Int	2009	snRNU6	human
Te/Ojwang	Lupus nephritis	Identification of unique microRNA signature associated with lupus nephritis	PLoS ONE	2010	snRNU6	human
**Bao/Liu**	**IgA-Nephropathy**	**MiR-223 downregulation promotes glomerular endothelial cell activation by upregulating importin α4 and α5 in IgA nephropathy**	**Kidney Int.**	**2013**	**snRNU6**	**human (Micro-dissection)**
**Bao/Liu**	**IgA-Nephropathy**	**Inhibition of miRNA-21 prevents fibrogenic activation in podocytes and tubular cells in IgA nephropathy**	**Biochem Biophys Research Com**	**2014**	**snRNU6**	**human (Micro-dissection)**
Cheng/ Singhal	HIVAN	MicroRNAs in HIV-associated nephropathy (HIVAN)	Exp Mol Pathol	2013	snRNU6	human/mouse
Long/Danesh	Diabetic Nephropathy	Identification of MicroRNA-93 as a Novel Regulator of Vascular Endothelial Growth Factor in Hyperglycemic Conditions	J. Biol. Chem	2010	snRNU6	mouse
Oba/Fujita	Renal Fibrosis	miR-200b precursor can ameliorate renal tubulointerstitial fibrosis.	PLoS One	2010	snRNU6	mouse
Zhong/ Lan	Renal Fibrosis	Smad3-Mediated Upregulation of miR-21 Promotes	J. Am. Soc. Nephrol	2011	snRNU6	mouse
Qin/Lan	Renal Fibrosis	TGF-β/Smad3 signaling promotes renal fibrosis by inhibiting miR-29	J. Am. Soc. Nephrol	2011	snRNU6	mouse
Park/ Natarajan	Diabetic Nephropathy	FOG2 protein down-regulation by transforming growth factor‑β1-induced microRNA‑200b/c leads to Akt kinase activation and glomerular mesangial hypertrophy related to diabetic nephropathy	J. Biol. Chem	2013	snRNU6	mouse
Mu/ Feng	Diabetic Nephropathy	Functional Implications of MicroRNA-215 in TGF-β1-Induced Phenotypic Transition of Mesangial Cells by Targeting CTNNBIP1	PLoS One	2013	snRNU6	mouse
Li/Lan	Renal Fibrosis	The microRNA miR-433 promotes renal fibrosis by amplifying the TGF-β/Smad3-Azin1 pathway	Kidney Int	2013	snRNU6	mouse
Jiang/Yang	Renal Fibrosis	A microRNA-30e/mitochondrial uncoupling protein 2 axis mediates TGF-β1-induced tubular epithelial cell extracellular matrix production and kidney fibrosis	Kidney Int.	2013	snRNU6	mouse
Wang/Yang	Diabetic Nephropathy	Tongxinluo ameliorates renal structure and function by regulating miR-21-induced epithelial-mesenchymal transition in diabetic nephropathy.	Am. J. Physiol. Renal Physiol	2014	snRNU6	mouse
Lin/Wang	Diabetic Nephropathy	MicroRNA-29a promotion of nephrin acetylation ameliorates hyperglycemia-induced podocyte dysfunction	J. Am. Soc. Nephrol.	2014	snRNU6	mouse
Chen/Lan	Diabetic Nephropathy	MicroRNA-29b inhibits diabetic nephropathy in db/db mice	Mol. Ther	2014	snRNU6	mouse
Denby/ Baker	Renal Fibrosis	MicroRNA-214 antagonism protects against renal fibrosis	J. Am. Soc. Nephrol	2014	snRNU6	mouse
Zhong/ Lan	Diabetic Nephropathy	miR-21 is a key therapeutic target for renal injury in a mouse model of type 2 diabetes	Diabetologia	2013	snRNU6	mouse/rat
Chung/ Lan	Renal Fibrosis	Smad7 suppresses renal fibrosis via altering expression of TGF-β/Smad3-regulated microRNAs	Mol Ther	2013	snRNU6	mouse/rat
Xiong/Yang	Renal Fibrosis	The miR-200 family regulates TGF-β1-induced renal tubular epithelial to mesenchymal transition through Smad pathway by targeting ZEB1 and ZEB2 expression	Am. J. Physiol. Renal Physiol	2012	snRNU6	rat
Li/ Lin	Diabetic Nephropathy	Curcumin ameliorates Podocytic adhesive capacity damage under mechanical stress by inhibiting miR-124 expression	Kidney Blood Press Res	2013	snRNU6	rat

Autor / Seniorautor	Topic	Titel	Journal	Year	Reference microRNA	Renal Tissue (species)
Sui/Huang	Renal Allograft Rejection	Microarray analysis of MicroRNA expression in acute rejection after renal transplantation	Transpl Immunol	2008	snRNU6	human
Long/Danesh	Diabetic Nephropathy	MicroRNA-29c is a signature microRNA under high glucose conditions that targets Sprouty homolog 1, and its in vivo knockdown prevents progression of diabetic nephropathy	J Biol Chem	2011	snRNU6	mouse
Serino/ Schena	Diabetic Nephropathy	Abnormal miR-148b expression promotes aberrant glycosylation of IgA1 in IgA nephropathy	J. Am. Soc. Nephrol	2012	snRNU6	mouse
Macconi/ Benigni	Renal Fibrosis	MicroRNA-324-3p promotes renal fibrosis and is a target of ACE inhibition.	J. Am. Soc. Nephrol	2012	snRNU6 (also TLDA)	mouse (Micro-dissection)
Chung/ Lan	Renal Fibrosis	miR-192 mediates TGF-β/Smad3-driven renal fibrosis.	J. Am. Soc. Nephrol	2010	snRNU6 (snRNU6, RNU48 for TLDA)	rat
Liu/ Lan	Renal Fibrosis	Disruption of Smad7 promotes ANG II-mediated renal inflammation and fibrosis via Sp1-TGF-β/Smad3-NF.κB-dependent mechanisms in mice	PLoS One	2013	snRNU6 / GAPDH	mouse
Shi/ Bottinger	Glomerular Kidney Disease	Smad2-dependent downregulation of miR-30 is required for TGF-β-induced apoptosis in podocytes	PLoS One	2013	snRNU6, 5s RNA	mouse
Patel/ Igarashi	Polycystic Kidney Disease	MicroRNAs regulate renal tubule maturation through modulation of Pkd1.	J. Am. Soc. Nephrol	2012	snRNU6, 5s RNA, 18s RNA	mouse
Deshpande/Natarajan	Diabetic Nephropathy	Transforming growth factor-β-induced cross talk between p53 and a microRNA in the pathogenesis of diabetic nephropathy	Diabetes	2013	snRNU6, RNU44, RNU48	human/mouse (Micro-dissection)
Sene/ Boer	Renal Fibrosis	Involvement of renal corpuscle microRNA expression on epithelial-to-mesenchymal transition in maternal low protein diet in adult programmed rats	PLoS One	2013	snRNU6, RNU87	rat
Zhdanova/ Skolnik	Podocytopathia	The inducible deletion of Drosha and microRNAs in mature podocytes results in a collapsing glomerulopathy	Kidney Int	2011	snRNU6, RNU87 (TLDA)	mouse
Wang/ Kantharidis	Renal Fibrosis	Suppression of microRNA-29 expression by TGF-β1 promotes collagen expression and renal fibrosis	J. Am. Soc. Nephrol.	2012	snRNU6B	mouse/rat
Alvarez/ Kiefer	Diabetic Nephropathy	Role of microRNA 1207-5P and its host gene, the long non-coding RNA Pvt1, as mediators of extracellular matrix accumulation in the kidney: implications for diabetic nephropathy	PLoS One	2013	snRNU6B, RNU44	human
Wang/ Kantharidis	Diabetic Nephropathy	E-cadherin expression is regulated by miR-192/215 by a mechanism that is independent of the profibrotic effects of transforming growth factor-beta.	Diabetes	2010	snRNU6B, Sno135, or RNU87	mouse
Lee/LaRusso	Polycystic Kidney Disease	MicroRNA15a modulates expression of the cell-cycle regulator Cdc25A and affects hepatic cystogenesis in a rat model of polycystic kidney disease	J Clin Invest	2008	tRNA met	rat

Supplementary Table 3 (see excel file)

All 762 C_q values are provided for further individual analysis. 240 analyzed (32%; 180 from Pool A and 60 from Pool B) and the 522 discarded miRNAs are shown in separate sheets. C_q values >32 are highlighted in orange and C_q values = 45 in purple.
